# Underexpression of Carbamoyl Phosphate Synthetase I as Independent Unfavorable Prognostic Factor in Intrahepatic Cholangiocarcinoma: A Potential Theranostic Biomarker

**DOI:** 10.3390/diagnostics13132296

**Published:** 2023-07-06

**Authors:** Khaa Hoo Ong, Yao-Yu Hsieh, Ding-Ping Sun, Steven Kuan-Hua Huang, Yu-Feng Tian, Chia-Lin Chou, Yow-Ling Shiue, Keva Joseph, I-Wei Chang

**Affiliations:** 1 Division of Gastroenterology & General Surgery, Department of Surgery, Chi Mei Medical Center, Tainan 710, Taiwan; pegfrancis@gmail.com (K.H.O.); sdp0127@gmail.com (D.-P.S.); 2 Department of Medical Technology, Chung Hwa University of Medical Technology, Tainan 717, Taiwan; clchou3@gmail.com; 3 Institute of Biomedical Sciences, National Sun Yat-sen University, Kaohsiung 804, Taiwan; shirley@imst.nsysu.edu.tw; 4 Division of Hematology and Oncology, Department of Internal Medicine, Shuang Ho Hospital, Taipei Medical University, Taipei 235, Taiwan; alecto39@gmail.com; 5 Division of Hematology and Oncology, Department of Internal Medicine, School of Medicine, College of Medicine, Taipei Medical University, Taipei 110, Taiwan; 6 Division of Urology, Department of Surgery, Chi Mei Medical Center, Tainan 710, Taiwan; cmh7530@mail.chimei.org.tw; 7 Department of Medical Science Industries, College of Health Sciences, Chang Jung Christian University, Tainan 711, Taiwan; 8 Division of Colon and Rectal Surgery, Department of Surgery, Chi Mei Medical Center, Tainan 710, Taiwan; van0112@hotmail.com; 9 Institute of Precision Medicine, National Sun Yat-sen University, Kaohsiung 804, Taiwan; 10 St. Jude Hospital, Vieux Fort LC12 201, Saint Lucia; keva.joseph@gmail.com; 11 Department of Pathology, School of Medicine, College of Medicine, Taipei Medical University, Taipei 110, Taiwan; 12 Department of Clinical Pathology, Wan Fang Hospital, Taipei Medical University, Taipei 116, Taiwan; 13 Department of Pathology, Taipei Medical University Hospital, Taipei 110, Taiwan; 14 Department of Pathology, Shuang Ho Hospital, Taipei Medical University, Taipei 235, Taiwan

**Keywords:** biomarker, carbamoyl phosphate synthetase I, CPS1, diagnostic, intrahepatic cholangiocarcinoma, prognosis, theranostic, urea cycle

## Abstract

Intrahepatic cholangiocarcinoma (IHCC) is the second most common malignant neoplasm of the liver. In spite of the increasing incidence worldwide, it is relatively rare in Western countries. IHCC is relatively common in Eastern and Southeastern Asia. Patients with IHCC are usually diagnosed at an advanced stage, therefore, the clinical outcome is dismal. Dysregulation of urea cycle metabolic enzyme expression is found in different types of cancers. Nevertheless, a comprehensive evaluation of genes related to the urea cycle (i.e., GO:0000050) has not been conducted in IHCC. By performing a comparative analysis of gene expression profiles, we specifically examined genes associated with the urea cycle (GO:0000050) in a publicly accessible transcriptomic dataset (GSE26566). Interestingly, *CPS1* was identified as the second most prominently down-regulated gene in this context. Tumor tissues of 182 IHCC patients who underwent curative-intent hepatectomy were enrolled. The expression level of CPS1 protein in our IHCC cohort was assessed by immunohistochemical study. Subsequent to that, statistical analyses were carried out to examine the expression of CPS1 in relation to various clinicopathological factors, as well as to assess its impact on survival outcomes. We noticed that lower immunoreactivity of CPS1 in IHCC was associated with tumor progression (pT status) with statistical significance (*p* = 0.003). CPS1 underexpression was not only negatively correlated to overall survival (OS), disease-specific survival (DSS), local recurrence-free survival (LRFS) and metastasis-free survival (MeFS) in univariate analysis but also an independent prognosticator to forecast poorer clinical outcome for all prognostic indices (OS, DSS, LRFS and MeFs) in patients with IHCC (all *p* ≤ 0.001). These results support that CPS1 may play a crucial role in IHCC oncogenesis and tumor progression and serve as a novel prognostic factor and a potential diagnostic and theranostic biomarker.

## 1. Introduction

Cholangiocarcinomas are primary malignant neoplasms arising in epithelial lining of biliary tracts. They can be divided into extrahepatic and intrahepatic counterparts. The latter is more common than the former. Intrahepatic cholangiocarcinoma (IHCC) is the second most common primary hepatic malignancy after hepatocellular carcinoma [1]. The global incidence is higher in Eastern and Southeastern Asia, especially in Thailand, where 85 per 100,000 cases are diagnosed per year [2]. Due to asymptomatic disease in the early stage and the difficulty of early diagnosis, patients with IHCC are usually diagnosed in advanced disease with no possibility for surgical resection [3]. Since surgical resection with free margin remains the only potential curative therapy, the clinical outcome of IHCC is dismal [4]. The prognosis is still discouraging for patients undergoing curative-intent surgery, with a five-year survival rate of about 20% to 35% [5]. Hence, the search for new targetable treatment for IHCC patients is urgent.

Dysregulation of urea cycle metabolic enzyme expression is found in different types of cancers [6]. Among them, carbamoyl phosphate synthetase I (CPS1) is the cardinal enzyme in ureagenesis in consideration of its anabolism in the first and rate-limiting step of the urea cycle [7]. Altered expression of the *CPS1* gene and/or CPS1 protein in different malignancies has been investigated recently. Overexpression of CPS1 is found in glioblastoma [8], ovarian carcinoma [9], urothelial carcinoma of the urinary bladder [10], lung adenocarcinoma [11,12], breast cancer [13] and colorectal cancer [14]. By contrast, CPS1 down-regulation is noted in hepatocellular carcinoma [15], gastric adenocarcinoma [16] and adenocarcinoma of the small intestine [17]. The genes related to the Gene Ontology term urea cycle (GO:0000050) have not been systemically evaluated in IHCC. By analysis of the open-access transcriptomic gene expression data of IHCC (GSE26566) from the Gene Expression Omnibus, National Center for Biotechnology Information (GEO, NCBI, Bethesda, MD, USA), we found that *CPS1* mRNA is one of the most significantly down-regulated genes involved in the urea cycle (GO:0000050). However, there has been no systemic investigation of CPS1 expression and its clinical significance and prognostic value in IHCC. Therefore, we conducted the current study.

## 2. Materials and Methods

### 2.1. Analysis of Gene Expression Profiles from Publicly Accessible Intrahepatic Cholangiocarcinoma Database

The transcriptomic profiles by array of intrahepatic cholangiocarcinoma (IHCC) (GSE26566) including 104 surgically resected IHCC and matched adjacent hepatic tissues from 59 patients from the Gene Expression Omnibus, National Center for Biotechnology Information (GEO, NCBI, Bethesda, MD, USA) [18] were downloaded for analysis. The downloaded raw data were evaluated by comparative analysis by GeneChip™ Human Genome U133 Plus 2.0 Array (Thermo Fisher Scientific, Waltham, MA, USA). We focused on the transcriptomic levels of genes related to Gene Ontology term urea cycle (GO:0000050). The expression degree of genes was computed by probe combinations, which were settled without preselection or filtering. Following that, genes meeting the criteria of a *p*-value less than 0.01 and a log_2_-transformed expression fold change greater than ±0.2 were chosen for subsequent analysis.

### 2.2. Study Cohort of Patients, Tumor Tissue Samples and Histopathological Evaluation

Between 1990 to 2010, adequate paraffin-embedded tissue blocks from 182 patients diagnosed with IHCCs at Chi Mei Medical Center and undergoing surgical resection were collected. All patients were surgically resectable whilst those with distant metastasis at diagnosis were excluded from the study. All patients were subject to regular clinical follow-up after surgery, with monitoring continuing until either their death or their last appointment. The tissue sections from the blocks were routinely stained with hematoxylin and eosin. The important pathological parameters, such as surgical margins, vascular invasion, histopathological variants and grading, were re-evaluated by a pathologist (I.-W.C.). The histopathological grade and subtypes were evaluated according to the latest edition of the World Health Organization (WHO) Classification of Digestive System Tumors [19]. Approvals were authorized by the Institutional Review Board of Chi Mei Medical Center (IRB09912003) and Joint Institutional Review Board of Taipei Medical University (N202304035).

### 2.3. Immunohistochemical Study and Interpretation

To assess the expression of the CPS1 protein, immunohistochemistry (IHC) was conducted using surgically resected formalin-fixed, paraffin-embedded (FFPE) blocks. The procedure involved cutting 4 μm sections from the blocks and placing them onto pre-coated glass slides. Deparaffinization of the slides was carried out using xylene, followed by rehydration with ethanol. Antigen retrieval was performed by heating the sections in a 10 mM citrate buffer (pH = 6) for 7 min using a microwave. Endogenous peroxidases were blocked using 3% H_2_O_2_. Subsequently, the slides were washed with TRIS-buffered saline (TBS) for 15 min and incubated with a primary antibody against CPS1 (rabbit polyclonal, dilution 1:500, Sigma-Aldrich, Burlington, MA, USA). The cytoplasmic immunoreactivity of CPS1 was evaluated using the H-score, calculated using the following equation: H-score = ΣPi (i + 1), where i represents the intensity of the stained tumor cells (ranging from 0 to 3+) and Pi represents the percentage of cytoplasmic immunoreactivity in tumor cells at different intensities. Low CPS1 expression was defined as having an H-score below the median of all scored cases.

### 2.4. Statistical Analysis

The association between CPS1 expression and various clinicopathological features was assessed using Pearson’s chi-squared (χ^2^) test. Survival analysis, including overall survival (OS), disease-specific survival (DSS), local recurrence-free survival (LRFS) and metastasis-free survival (MeFS), was conducted using Kaplan–Meier plots. Univariate survival analyses were compared using log-rank tests. For the multivariate analysis, a Cox proportional hazards model was employed to identify independent prognostic factors. Statistical significance was defined as a *p*-value less than 0.05 using two-sided tests. All statistical analyses were performed using IBM SPSS Statistics 22.0 (IBM Corporation, Armonk, NY, USA).

## 3. Result

### 3.1. Urea Cycle-Associated Gene CPS1 Is Significantly Down-Regulated in Intrahepatic Cholangiocarcinomas Compared with Non-Cancerous Counterparts

**Figure 1:**
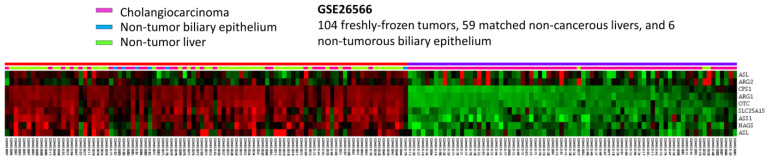
Analysis of gene expression in intrahepatic cholangiocarcinoma by utilizing a previous publicly accessible transcriptomic dataset (GSE26566). Through a clustering analysis of genes specifically associated with the urea cycle (GO:0000050), it was observed that *CPS1* is among the genes exhibiting the most significant down-regulation in cholangiocarcinoma when compared to non-tumor tissue. The heat map depicts cholangiocarcinoma (represented in pink), normal biliary epithelium (represented in blue) and normal liver (represented in green) at the top. The degree of up-regulation and down-regulation of gene expression is depicted by varying shades of red and green, respectively, with unchanged transcriptional levels indicated in black.

To develop the potential prognostic and diagnostic biomarkers and therapeutic targets for IHCC patients, the public intrahepatic cholangiocarcinoma (IHCC) transcriptomic dataset (GSE26566) from GEO, NCBI, comprising 104 IHCC cancer tissues, 59 non-tumorous hepatic tissues and 9 normal intrahepatic bile duct tissues, was downloaded. By data mining and focus on genes linked to Gene Ontology term urea cycle (GO:0000050), ten probes covering eight mRNA transcripts were identified (Figure 1). As shown in Figure 1 and Table 1, by comparing IHCC to non-tumorous hepatic tissues, seven mRNA transcripts are significantly down-regulated, including *ARG1*, *CPS1*, *OTC*, *SLC25A15*, *ASS1*, *ASL* and *NAGS* genes (*p* ≤ 0.036). By comparing IHCC to normal intrahepatic bile duct tissues, four transcripts are significantly down-regulated, including *ARG1*, *CPS1*, *OTC* and *SLC25A15* genes (*p* ≤ 0.0014). Among them, *ARG1* and *CPS1* genes exhibit the most significant down-regulation, whose log_2_ ratios by comparison between IHCC and non-tumor, as well as IHCC and normal bile duct, are −4.0505 and −2.9184 (*p* ≤ 0.0001) and −3.9435 and −2.8787 (*p* ≤ 0.0002), respectively. Taken together, these findings demonstrated that *ARG1* and *CPS1* gene alterations might play an essential role in IHCC cancer progression. However, the expression level and prognostic value of ARG1 in IHCC have been comprehensively evaluated [20]. In addition, arginase-1, encoded by the *ARG1* gene, is also considered as a sensitive and specific immunohistochemical marker of normal cells and benign and malignant neoplasms derived from hepatocytes [21]. Therefore, we focused on the expression of CPS1.

### 3.2. CPS1 Expression and the Associations with Clinical and Pathological Variables of Cholangiocarcinoma Patients

**Figure 2:**
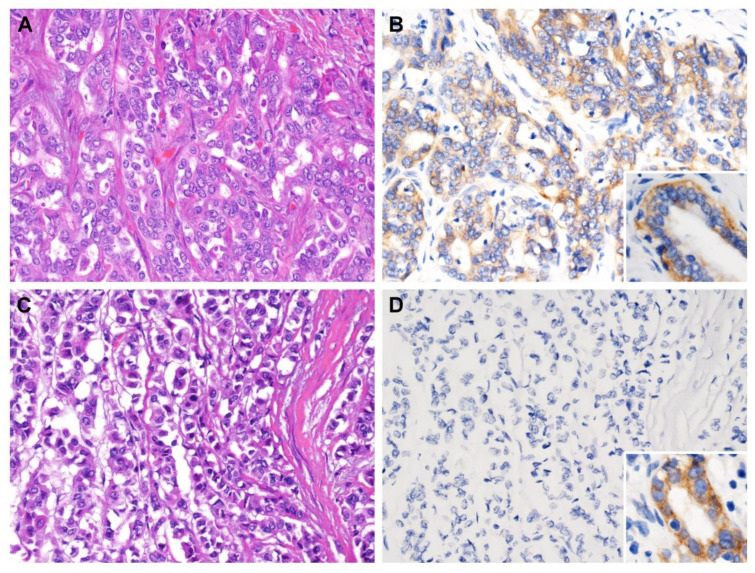
CPS1 immunostaining of representative sections. (**A**) Solitary tumor without vascular invasion (pT1) exhibited (**B**) strong cytoplasmic immunoreactivity, while (**C**) tumor with visceral peritoneum perforation (pT3) was (**D**) negative for CPS1 immunostain. Note the strong expression in normal biliary epithelium (inset). ((**A**,**C**) hematoxylin and eosin stain, magnification 200×; (**B**,**D**) CPS1 immunostain, magnification 200×).

The aforementioned data suggested that low expression of CPS1 may be interrelated with carcinogenesis and tumor progression of IHCC. Accordingly, the correlation between CPS1 expression and the clinical and pathological features of IHCC patients was further scrutinized. The details of our patient cohort are revealed in Table 2. In total, we collected 182 cases of patients with primary localized IHCC, and 108 were male and 74 were female (M:F = 59.3%:40.7%), of which 41.2% patients (*n* = 75) were over 65 years old and 58.8% patients (*n* = 107) were younger than 65 years. Eighty patients (44.0%) had intrahepatic cholelithiasis. The resection margins of 19 IHCCs were positive (R1 resection, 10.4%). Histopathologically, the majority (57.7%, *n* = 105) were large duct type, while 42.3% of cases (*n* = 77) were small duct type. Sixty-one cases (33.5%) were grade 1 (well differentiated), sixty-six (36.3%) were grade 2 (moderately differentiated) and fifty-five (30.2%) were grade 3 (poorly differentiated). Furthermore, we analyzed clinicopathological parameters and found that lower expression of CPS1 in IHCCs was prominently related to more advanced primary tumor (pT1, pT2 and pT3) with statistical significance (Figure 2, *p* = 0.003). On the other hand, the expression of CPS1 was not significantly associated with gender, age, types of hepatitis, intrahepatic stones, surgical margins and histological variants and grading.

### 3.3. Survival Analyses for Patients with Intrahepatic Cholangiocarcinoma

The survival analyses are presented in Table 3 and Table 4. In the univariate log-rank test, male patients had better overall survival (OS) and disease-specific survival (DSS) compared to female patients (*p* = 0.0254 and 0.0072, respectively). Free surgical margin after hepatectomy (R0 resection) and less-advanced cancer disease (pT status) were positively linked to longer overall survival (OS), disease-specific survival (DSS), local recurrence-free survival (LRFS) and metastasis-free survival (MeFS) intervals (all *p* ≤ 0.0001). Small duct type and lower histological grade were significantly associated with improved LRFS rate (*p* = 0.0085 and 0.0299, respectively). In the multivariate analysis, only surgical margin was an independent prognostic factor for all survival indices, i.e., OS (relative risk = 3.013, 95% confidence interval = 1.517–5.985, *p* = 0.002), DSS (RR = 5.639, 95% CI = 2.472–12.863, *p* < 0.001), LRFS (RR = 4.209, 95% CI = 2.122–8.348, *p* < 0.001) and MeFS (RR = 3.034, 95% CI = 1.470–6.260, *p* = 0.003). Extension of primary tumor (pT status) was an independent indicator for DSS, LRFS and MeFS (*p* = 0.016, 0.041 and 0.032, respectively) but not OS (*p* = 0.055).

### 3.4. CPS1 Expression as an Independent Prognosticator in Patients with Intrahepatic Cholangiocarcinoma

**Figure 3:**
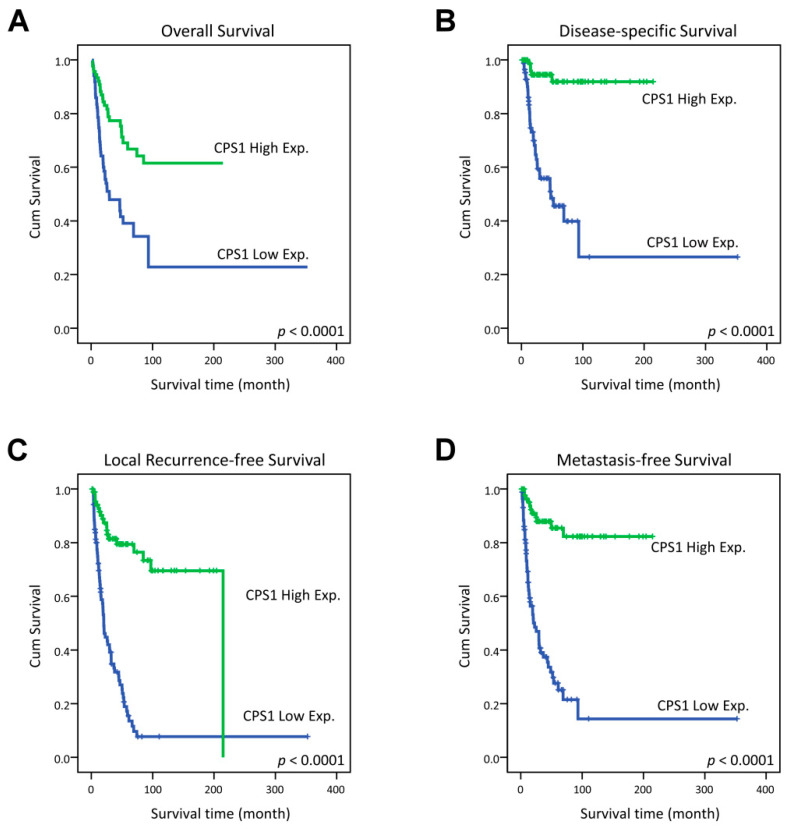
Kaplan–Meier estimator demonstrated the significantly poorer clinical outcomes, including (**A**) overall survival (OS), (**B**) disease-specific survival (DSS), (**C**) local recurrence-free survival (LRFS) and (**D**) distant metastasis-free survival (MeFS) in relation to the low expression of CPS1 (all *p* < 0.0001).

In the univariate analysis, CPS1 underexpression was significantly linked to poorer clinical outcome of all survival indices, including OS, DSS, LRFS and MeFS (all *p* < 0.0001, Figure 3). Remarkably, low expression of CPS1 even independently predicted adverse OS (relative risk = 2.378, 95% confidence interval = 1.424–3.971, *p* = 0.001), DSS (RR = 9.957, 95% CI = 3.817–25.975, *p* < 0.001), LRFS (RR = 5.519, 95% CI = 3.214–9.477, *p* < 0.001) and MeFS (RR = 7.417, 95% CI = 3.814–14.422, *p* < 0.001) in multivariate analysis (Table 3 and Table 4).

## 4. Discussion

Intrahepatic cholangiocarcinoma (IHCC), also known as bile duct carcinoma, is the second most common primary cancer of the liver. It accounts for about 10–15% of primary hepatic malignancies [22]. It is relatively rare in Western countries, however, it is more common in Southeastern Asia owing to endemic liver fluke infection, e.g., *Opisthorchis viverrini* [23]. Histologically, it is divided into two main subtypes: large duct and small duct types [24]. The risk factors differ between these two subtypes. Large duct IHCC exhibits similar risk factors to extrahepatic and perihilar cholangiocarcinoma, including liver fluke infection, biliary lithiasis, primary sclerosing cholangitis, Caroli disease, etc., while small duct IHCC shares the same risk factors as hepatocellular carcinoma, such as chronic hepatitis B and C, alcoholic and non-alcoholic steatohepatitis, as well as non-biliary cirrhosis [25]. Generally, both large duct and small duct types of IHCC are associated with chronic inflammation of the biliary tree and cholestasis [26]. In spite of the improvement of treatment modality, the prognosis of IHCC is still dismal [27]. Five-year survival rate and overall survival after surgical excision range from 15% to 40% for resectable cases [27]. Accordingly, the search for new targetable treatment for IHCC patients is critical.

The urea cycle, also called the ornithine cycle, was first discovered by Hans Krebs and Kurt Henseleit in 1932 [28,29,30]. Ordinarily, the urea cycle primarily takes place in the liver [31]. It is a critical biochemical reaction for mammals to convert highly toxic ammonia to urea for elimination and also the principal route to excrete excess nitrogen in ureotelic animals, including humans. Urea cycle dysregulation is commonly found in different cancer types. In contrast with nitrogen disposal in normal hepatocytes, cancer cells tend to redirect urea cycle intermediates to anabolic pathways. An aberrance results in reduced production of nitrogen waste and increased alteration of carbon and nitrogen biosynthesis to fulfill nutrition demand of cancer cells and their growth [6]. Abnormal utilization of nitrogen in cancer cells also leads to increased synthesis of pyrimidine and subsequent nucleotide pool imbalance and transversion mutations [32].

There are six enzymes involved in the urea cycle, including carbamoyl phosphate synthetase I (CPS1), N-acetylglutamate synthase (NAGS), ornithine transcarbamylase (OTC), argininosuccinate synthase (ASS1), argininosuccinate lyase (ASL) and arginase-1 (ARG1). Among them, CPS1 administers the first step and the rate-limiting reaction of the urea cycle. CPS1 catalyzes transfer of ammonia to phosphorylated bicarbonate with the production of carbamoyl phosphate [33]. Afterward, carbamoyl phosphate enters the urea cycle. Normally, the enzyme CPS1 is predominantly located in the mitochondria of the hepatocytes and is also detected in the mucosa of the small bowel [34]. Diseases associated with genetic alteration of the *CPS1* gene have been discovered. For example, germline mutation of the *CPS1* gene causes CPS1 deficiency, a rare lethal urea cycle disorder with autosomal recessive inheritance, causing the death of newborns owing to hyperammonemia [35].

There has been increasing evidence that CPS1 is involved in tumorigenesis. In 2002, Kinoshita et al. firstly identified a down-regulated transcriptomic level of the *CPS1* gene in 15 out of 20 (75%) human hepatocellular carcinoma (HCC) tissues compared with adjacent non-cancerous hepatitis tissues [15]. After that, Cao et al. also discovered that CPS1 was significantly underexpressed in HCCs with macrovascular invasion by an iTRAQ-based proteomic study [36]. The mechanism of dysregulated CPS1 in HCCs may contribute to hypermethylation of the two CpG sites of the promoter of the *CPS1* gene [37]. In the study of gastric carcinogenesis by Fang et al., the immunoreactivity of CPS1 protein reveals gradually lower expression from intestinal metaplasia, low-grade dysplasia and high-grade dysplasia to intestinal-type gastric adenocarcinoma [16]. In the same research, lower expression of CPS1 was also significantly associated with unfavorable overall survival in both univariate and multivariate analyses [16]. Similarly, we identified *CPS1* as one of the highly down-regulated genes associated with the Gene Ontology term “urea cycle” (GO:0000050) by analysis of the open-access transcriptomic gene expression data of IHCC (GSE26566). Lower immunoreactivity of CPS1 in IHCC was associated with tumor progression (pT status) with statistical significance (*p* = 0.003) in the current study. Furthermore, CPS1 underexpression was not only negatively correlated to overall survival (OS), disease-specific survival (DSS), local recurrence-free survival (LRFS) and metastasis-free survival (MeFS) in univariate analysis but also an independent prognosticator to forecast poorer clinical outcome for all prognostic indices (OS, DSS, LRFS and MeFs) in patients with IHCC (all *p* ≤ 0.001). Conversely, up-regulation of the *CPS1* gene and overexpression of CPS1 protein are described in glioblastoma and carcinomas of the urinary bladder, ovary, lung and colon [8,9,10,11,12,13,14]. Overexpression of CPS1 is usually associated with adverse clinical outcome in patients with these cancer types. The discrepancies of CPS1 expression between two groups of cancers may be due to different mechanisms in tumorigenesis and tumor progression. In the former cancers, the low expression of CPS1 leads to exaggerated production of ammonia, which may result in reactive oxygen species (ROS) creation, adenosine monophosphate-activated protein kinase (AMPK) phosphorylation and hyperactivation of the AMPK–fatty acid oxidation (FAO)–forkhead box protein M1 (FOXM1) axis. Finally, fatty acid oxidation (FAO) supplies plentiful ATP for cancer cell proliferation [38]. In the latter cancer cells, overexpression of CPS1 increases nucleotide synthesis, as well as increases the pyrimidine to purine ratio, which may induce progression of the S phase of the cell cycle [11,39].

Over the past few years, a significant number of long non-coding RNAs (lncRNAs) have been discovered as essential contributors to the progression of different cancers, including IHCC [40]. In 2015, Ma et al. observed that the *CPS1* transcript and its long non-coding RNA, CPS1 intronic transcript 1 (CPS1-IT1), were co-up-regulated in IHCC tissue from 31 patients compared with paired non-tumorous tissues, as well as in an IHCC cell line (ICC-9810) compared with a human fetal hepatocyte line (L-02) [41]. In addition, the authors addressed that 4-fold/5-fold up-regulation of the *CPS1* transcript in IHCCs compared with benign counterparts was significantly correlated to worse disease-free survival and overall survival by a univariate log-rank test (*p* = 0.034 and 0.032, respectively) [41]. However, the difference in ΔCt values of CPS1 mRNA was not significantly lower in patients’ IHCC tissue (*p* = 0.09) and the IHCC cell line (*p* = 0.05). Moreover, multivariate survival analysis was not performed in that study. The divergent results of the investigation of Ma et al. compared to the current study may also have arisen from the intricate transcription and translation processes, leading to variations in mRNA and protein expression levels.

Small-molecule inhibitors of CPS1 have been discovered recently [42,43]. In the initial stage of carbamoyl phosphate synthesis, small-molecule inhibitors of CPS1 bind to an allosteric pocket, effectively inhibiting ATP hydrolysis. These CPS1 inhibitors have demonstrated activity in cellular assays by blocking both urea synthesis and the support provided by CPS1 to the pyrimidine biosynthetic pathway [42]. These discoveries suggest potential theranostic implications of small-molecule compounds targeting CPS1.

## 5. Conclusions

To the best of our knowledge, the current study is the first one systemically evaluating the relationship between CPS1 expression and the important clinicopathological parameters and clinical outcomes in a large intrahepatic cholagiocarcinoma (IHCC) patient cohort. We mined the public-access transcriptomic database and identified *CPS1* as one of the most significant down-regulated genes in IHCCs compared with normal counterparts. Furthermore, we recognized that underexpression of CPS1, the rate-limiting enzyme that regulates the first reaction of the urea cycle, was not only significantly associated with tumor progression (pT status) of IHCC but also an independent prognosticator predicting lower overall survival (OS), disease-specific survival (DSS), local recurrence-free survival (LRFS) and metastasis-free survival (MeFS) rate in patients with IHCC. It may serve as a novel prognostic factor and potential theranostic biomarker for patients with IHCC. Further investigations to clarify the complete molecular mechanisms of CPS1 in the oncogenesis of IHCC are obligatory for developing a promising CPS1-targeting therapy for high-risk patients.

## Figures and Tables

**Table 1:** Summary of the alterations of genes associated with urea cycle (GO:0000050) in cholangiocarcinoma (GSE26566).

Probe	CCA vs. Non-Tumor ^#^		CCA vs. Normal Intrahepatic Bile Duct ^&^		Gene Symbol	Molecular Function	Biological Process
	Log Ratio	*p*-Value	Log Ratio	*p*-Value			
ILMN_1812281	−4.0505	0 *	−2.9184	0.0001 *	*ARG1*	Metal ion binding, hydrolase activity, arginase activity, manganese ion binding	Arginine catabolism, urea cycle
ILMN_1792748	−3.9435	0 *	−2.8787	0.0002 *	*CPS1*	Ligase activity, nucleotide binding, ATP binding, carbamoyl phosphate synthase (ammonia) activity, protein binding	Nitrogen compound metabolism, pyrimidine base biosynthesis, urea cycle, arginine biosynthesis, glutamine metabolism
ILMN_1749114	−3.27	0 *	−1.8575	0.0013 *	*OTC*	Transferase activity, amino acid binding, ornithine carbamoyltransferase activity	Amino acid biosynthesis, urea cycle, arginine biosynthesis
ILMN_1667670	−1.8491	0 *	−1.1629	0.0014 *	*SLC25A15*	L-ornithine transporter activity, transporter activity, binding	Transport, mitochondrial ornithine transport, amino acid metabolism, urea cycle
ILMN_1708778	−2.0977	0 *	−0.7505	0.1192	*ASS1*	Ligase activity, nucleotide binding, ATP binding, protein binding, argininosuccinate synthase activity	Amino acid biosynthesis, urea cycle, arginine biosynthesis
ILMN_1800898	−0.1432	0.104	−0.1602	0.5411	*ARG2*	Metal ion binding, hydrolase activity, arginase activity, manganese ion binding	Nitric oxide biosynthesis, arginine catabolism, urea cycle
ILMN_1688234	−0.0856	0.036 *	−0.14	0.1737	*ASS1*	Ligase activity, nucleotide binding, ATP binding, protein binding, argininosuccinate synthase activity	Amino acid biosynthesis, urea cycle, arginine biosynthesis
ILMN_1685142	−0.1229	0.0015 *	−0.091	0.3739	*ASL*	Lyase activity, argininosuccinate lyase activity, catalytic activity	Arginine catabolism, amino acid biosynthesis, urea cycle, arginine biosynthesis
ILMN_1685037	−0.6339	0 *	−0.0032	0.9827	*ASL*	Lyase activity, argininosuccinate lyase activity, catalytic activity	Arginine catabolism, amino acid biosynthesis, urea cycle, arginine biosynthesis
ILMN_1758597	−0.6543	0 *	0.0218	0.894	*NAGS*	Acyltransferase activity, transferase activity, amino acid N-acetyltransferase activity, acetylglutamate kinase activity	Amino acid biosynthesis, urea cycle, arginine biosynthesis

**^#^**, comparing cholangiocarcinoma (CCA, *n* = 104) to surrounding liver (*n* = 59) and normal intrahepatic bile duct (*n* = 6); ^&^, comparing cholangiocarcinoma (CCA, *n* = 104) to normal intrahepatic bile duct (*n* = 6); * statistically significant.

**Table 2:** Correlations between CPS1 expression and other important clinicopathological parameters in primary localized IHCC.

Parameter	Category	Case No.	CPS1 Expression		*p*-Value
			Low	High	
Gender	Male	108	54	54	0.591
	Female	74	34	40	
Age (years)	<65	107	57	50	0.113
	≥65	75	31	44	
Hepatitis	Hepatitis B	72	37	35	0.458
	Hepatitis C	29	11	18	
	Non-B, non-C	81	40	41	
Intrahepatic lithiasis	Not identified	102	45	57	0.197
	Present	80	43	37	
Surgical margin	R0	163	76	87	0.172
	R1	19	12	7	
Primary tumor (T)	T1	87	32	55	0.003 *
	T2	61	32	29	
	T3	34	24	10	
Histological variants	Large duct type	105	56	49	0.116
	Small duct type	77	32	45	
Histological grade	Well differentiated	61	27	34	0.660
	Moderately differentiated	66	32	34	
	Poorly differentiated	55	29	26	

* Statistically significant.

**Table 3:** Univariate log-rank and multivariate analyses for overall and disease-specific survivals in primary localized IHCC.

Parameter	Category	Case No.	Overall Survival					Disease-Specific Survival				
			Univariate Analysis		Multivariate Analysis			Univariate Analysis		Multivariate Analysis		
			No. of Event	*p*-Value	R.R.	95% C.I.	*p*-Value	No. of Event	*p*-Value	R.R.	95% C.I.	*p*-Value
Gender	Male	108	50	0.0254 *	1	-	0.080	9	0.0072 *	1	-	0.050
	Female	74	21		1.587	0.946–2.662	-	32		2.141	1.001–4.582	-
Age (years)	<65	107	37	0.2626	-	-	-	28	0.2125	-	-	-
	≥65	75	34		-	-	-	13		-	-	-
Hepatitis	Hepatitis B	72	32	0.2379	-	-	-	16	0.4561	-	-	-
	Hepatitis C	29	8		-	-	-	19		-	-	-
	Non-B, non-C	81	31		-	-	-	6		-	-	-
Intrahepatic lithiasis	Not identified	102	36	0.2831	-	-	-	19	0.1613	-	-	-
	Present	80	35		-	-	-	22		-	-	-
Surgical margin	R0	163	59	<0.0001 *	1	-	0.002 *	31	<0.0001 *	1	-	<0.001 *
	R1	19	12		3.013	1.517–5.985		10		5.639	2.472–12.863	
Primary tumor (T)	T1	87	25	0.0001 *	1	-	0.055	9	<0.0001 *	1	-	0.016 *
	T2	61	27		1.678	0.966–2.915	-	19		2.679	1.102–6.514	-
	T3	34	19		2.101	1.102–4.006	-	13		3.185	1.424–7.121	-
Histological variants	Large duct type	105	43	0.4281	-	-	-	27	0.1984	-	-	-
	Small duct type	77	28		-	-	-	14		-	-	-
Histological grade (differentiation)	Well	61	20	0.1663	-	-	-	12	0.3881	-	-	-
	Moderately	66	28		-	-	-	16		-	-	-
	Poorly	55	23		-	-	-	13		-	-	-
CPS1 expression	High expression	94	25	<0.0001 *	1	-	0.001 *	5	<0.0001 *	1	-	<0.001 *
	Low expression	88	46		2.378	1.424–3.971	-	36		9.957	3.817–25.975	-

C.I.: Confidence Interval; R.R.: Relative Risk; * statistically significant.

**Table 4:** Univariate log-rank and multivariate analyses for local recurrence-free and metastasis-free survivals in primary localized IHCC.

Parameter	Category	Case No.	Local Recurrence-Free Survival					Metastasis-Free Survival				
			Univariate Analysis		Multivariate Analysis			Univariate Analysis		Multivariate Analysis		
			No. of Event	*p*-Value	R.R.	95% C.I.	*p*-Value	No. of Event	*p*-Value	R.R.	95% C.I.	*p*-Value
Gender	Male	108	54	0.2170	-	-	-	21	0.1008	-	-	-
	Female	74	31		-	-	-	44		-	-	-
Age (years)	<65	107	55	0.2993	-	-	-	42	0.2936	-	-	-
	≥65	75	30		-	-	-	23		-	-	-
Hepatitis	Hepatitis B	72	33	0.7333	-	-	-	26	0.8762	-	-	-
	Hepatitis C	29	13		-	-	-	11		-	-	-
	Non-B, non-C	81	39		-	-	-	28		-	-	-
Intrahepatic lithiasis	Not identified	102	41	0.0551	-	-	-	31	0.1000	-	-	-
	Present	80	44		-	-	-	34		-	-	-
Surgical margin	R0	163	71	<0.0001 *	1	-	<0.001 *	54	<0.0001 *	1		0.003 *
	R1	19	14		4.209	2.122–8.348		11		3.034	1.470–6.260	
Primary tumor (T)	T1	87	28	<0.0001 *	1	-	0.041 *	21	<0.0001 *	1	-	0.032 *
	T2	61	32		1.797	1.028–3.143		26		2.055	1.145–3.689	
	T3	34	25		2.099	1.142–3.858		18		2.049	1.058–3.968	
Histological variants	Large duct type	105	58	0.0085 *	1	-	0.571	43	0.0759	-	-	-
	Small duct type	77	27		0.868	0.532–1.417		22		-	-	-
Histological grade (differentiation)	Well	61	28	0.0299 *	1	-	0.568	22	0.1794	-	-	-
	Moderately	66	27		0.854	0.495–1.473		22		-	-	-
	Poorly	55	30		1.610	0.670–2.011		21		-	-	-
CPS1 expression	High expression	94	19	<0.0001 *	1	-	<0.001 *	11	<0.0001 *	1	-	<0.001 *
	Low expression	88	66		5.519	3.214–9.477		54		7.417	3.814–14.422	

C.I.: Confidence Interval; R.R.: Relative Risk; * Statistically significant.

## Data Availability

The data presented in this study are available on request from the corresponding author. The data are not publicly available due to privacy and ethical reasons.
