# Correction: Ong et al. Underexpression of Carbamoyl Phosphate Synthetase I as Independent Unfavorable Prognostic Factor in Intrahepatic Cholangiocarcinoma: A Potential Theranostic Biomarker. *Diagnostics* 2023, *13*, 2296

**DOI:** 10.3390/diagnostics15030391

**Published:** 2025-02-06

**Authors:** Khaa Hoo Ong, Yao-Yu Hsieh, Ding-Ping Sun, Steven Kuan-Hua Huang, Yu-Feng Tian, Chia-Lin Chou, Yow-Ling Shiue, Keva Joseph, I-Wei Chang

**Affiliations:** 1 Division of Gastroenterology & General Surgery, Department of Surgery, Chi Mei Medical Center, Tainan 710, Taiwan; pegfrancis@gmail.com (K.H.O.); sdp0127@gmail.com (D.-P.S.); 2 Department of Medical Technology, Chung Hwa University of Medical Technology, Tainan 717, Taiwan; clchou3@gmail.com; 3 Institute of Biomedical Sciences, National Sun Yat-sen University, Kaohsiung 804, Taiwan; shirley@imst.nsysu.edu.tw; 4 Division of Hematology and Oncology, Department of Internal Medicine, Shuang Ho Hospital, Taipei Medical University, Taipei 235, Taiwan; alecto39@gmail.com; 5 Division of Hematology and Oncology, Department of Internal Medicine, School of Medicine, College of Medicine, Taipei Medical University, Taipei 110, Taiwan; 6 Division of Urology, Department of Surgery, Chi Mei Medical Center, Tainan 710, Taiwan; cmh7530@mail.chimei.org.tw; 7 Department of Medical Science Industries, College of Health Sciences, Chang Jung Christian University, Tainan 711, Taiwan; 8 Division of Colon and Rectal Surgery, Department of Surgery, Chi Mei Medical Center, Tainan 710, Taiwan; van0112@hotmail.com; 9 Institute of Precision Medicine, National Sun Yat-sen University, Kaohsiung 804, Taiwan; 10 St. Jude Hospital, Vieux Fort LC12 201, Saint Lucia; keva.joseph@gmail.com; 11 Department of Pathology, School of Medicine, College of Medicine, Taipei Medical University, Taipei 110, Taiwan; 12 Department of Clinical Pathology, Wan Fang Hospital, Taipei Medical University, Taipei 116, Taiwan; 13 Department of Pathology, Taipei Medical University Hospital, Taipei 110, Taiwan; 14 Department of Pathology, Shuang Ho Hospital, Taipei Medical University, Taipei 235, Taiwan

In the original publication [1], there was a mistake in Figure 3B as published. The group with the poorer outcome in Figure 3B should have been labeled “CPS1 Low Exp”. The corrected Figure 3 appears below.

There was an error in the original publication. “Disease-free survival (DFS)” should be replaced with “disease-specific survival (DSS)”.

A correction has also been made to the Abstract:

CPS1 underexpression was not only negatively correlated to overall survival (OS), disease-specific survival (DSS), local recurrence-free survival (LRFS) and metastasis-free survival (MeFS) in univariate analysis but also an independent prognosticator to forecast poorer clinical outcome for all prognostic indices (OS, DSS, LRFS and MeFs) in patients with IHCC (all *p* ≤ 0.001).

A correction has also been made to the Discussion section, Paragraph 4:

Furthermore, CPS1 underexpression was not only negatively correlated to overall survival (OS), disease-specific survival (DSS), local recurrence-free survival (LRFS) and metastasis-free survival (MeFS) in univariate analysis but also an independent prognosticator to forecast poorer clinical outcome for all prognostic indices (OS, DSS, LRFS and MeFs) in patients with IHCC (all *p* ≤ 0.001).

The authors state that the scientific conclusions are unaffected. This correction was approved by the Academic Editor. The original publication has also been updated.

## Figures and Tables

**Figure 3:**
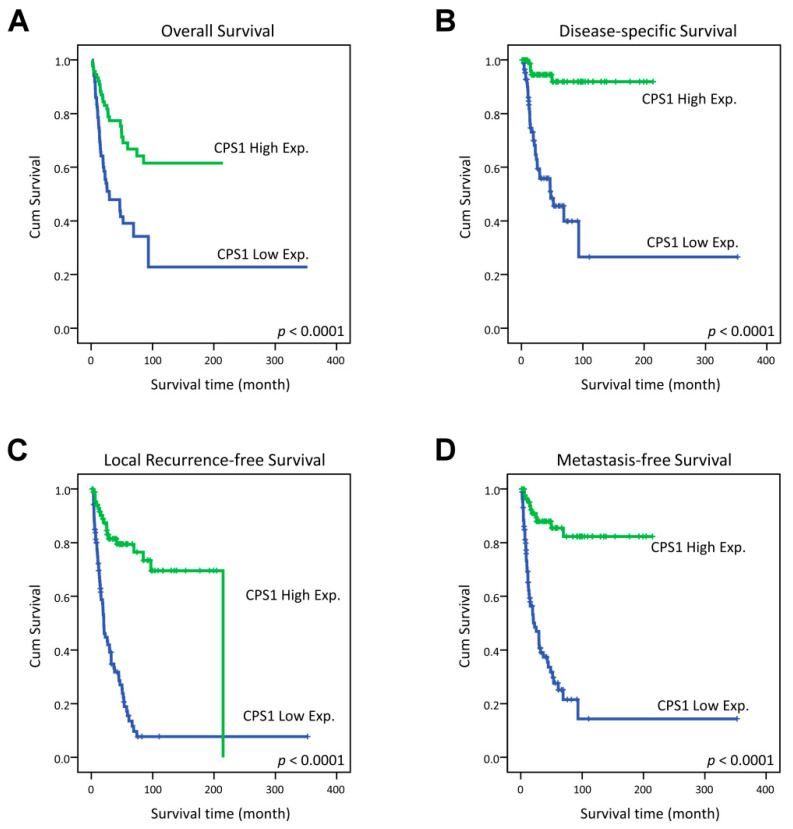
Kaplan–Meier estimator demonstrated the significantly poorer clinical outcomes, including (**A**) overall survival (OS), (**B**) disease-specific survival (DSS), (**C**) local recurrence-free survival (LRFS) and (**D**) distant metastasis-free survival (MeFS) in relation to the low expression of CPS1 (all *p* < 0.0001).
